# Aluminum V. Vulcanite. An Improvement in Artificial Teeth

**Published:** 1868-06

**Authors:** Sam'l R. Percy

**Affiliations:** Professor of Materia Medica. New York


ALUMINUM *v.* VULCANITE. AN IMPROVEMENT IN ARTIFICIAL TEETH.—Dr. Alfred Starr, of New York city, has brought for my inspection and chemical examination a set of teeth fastened upon a plate of aluminum, together with some solder for uniting the teeth to the aluminum plate. I have made a chemical examination of this solder, and find that there is neither mercury, arsenic, zinc, or lead in its composition, but that it is composed of a metal that is perfectly harmless in the mouth.

I consider this use of aluminum one of the most scientific improvements in dentistry. Aluminum is the metal of which alum is a salt. For dentistry it is infinitely preferable to gold, being both lighter and cheaper. Between the use of aluminum and hard rubber there can be no comparison; for while aluminum is strong, and the lightest of metals, and perfectly harmless in the mouth; hard rubber is thick, heavier, brittle, and exceedingly dangerous, there being many instances of mercurial salivation attributed to its use.

The hard rubber used for teeth base is composed of rubber, sulphur and sulphide of mercury, nearly one-third of the whole being sulphide of mercury. This amount of mercury kept constantly in the mouth, cannot but be sometimes injurious, as it is soluble in the saliva.

In addition to the danger of salivation from the mercury in these hard rubber plates there are other dangers unknown to the manufacturer. The sulphide of mercury which is added to the rubber is often adulterated with red lead and bi-sulphide of arsenic, both of which poisons are soluble in the mouth. By inserting this in your valuable journal, you will be benefiting the public. SAM'L R. PERCY, M. D.,

*Professor of Materia Medica.*

NEW YORK, May, 1868.

